# Herbal *HuoXueTongFu* Formula with anti-inflammatory and fibrinolytic activity regulation for the prevention of postoperative peritoneal adhesions

**DOI:** 10.3389/fimmu.2024.1510766

**Published:** 2025-01-23

**Authors:** Lili Yang, Yali Wang, Zhengjun Li, Wen Li, Yanqi Chen, Ziyang Kong, Huixiang Zhang, Jiafei Wu, Mingqi Shang, Ming Li, Yaoyao Bian, Li Zeng

**Affiliations:** ^1^ Jiangsu Provincial Engineering Research Center of TCM External Medication Development and Application, Nanjing University of Chinese Medicine, Nanjing, China; ^2^ Jingwen Library, Nanjing University of Chinese Medicine, Nanjing, China; ^3^ School of First Clinical Medicine, Nanjing University of Chinese Medicine, Nanjing, China; ^4^ Department of Proctology, the First Affiliated Hospital of Anhui University of Chinese Medicine, Hefei, China; ^5^ College of Health Economics Management, Nanjing University of Chinese Medicine, Nanjing, China; ^6^ School of Health Preservation and Rehabilitation, Nanjing University of Chinese Medicine, Nanjing, China; ^7^ TCM Rehabilitation Center, Jiangsu Second Chinese Medicine Hospital, Nanjing, China; ^8^ Faculty of Chinese Medicine, Macau University of Science and Technology, Macao, Macao SAR, China

**Keywords:** *HuoXueTongFu* formula, postoperative peritoneal adhesion, macrophage polarization, fibrinolytic activity, time window

## Abstract

**Background:**

The *HuoXueTongFu* Formula (HXTF) originates from the classic prescription “*DaHuangMuDan* Decoction” from the “Synopsis of the golden chamber”. Our previous study revealed that HXTF has a positive effect on postoperative peritoneal adhesion (PPA). However, the specific mechanism of HXTF on PPA formation within the time-to-treatment window has not been fully elucidated. This study aimed to determine the critical roles of HXTF as a result of its specific anti-inflammatory and antifibrinolytic activities for PPA treatment.

**Methods:**

The eight main bioactive components of HXTF were subjected to high-performance liquid chromatography-mass spectrometry. The core targets, critical biological processes, and underlying pathways of HXTF and PPA were identified via a series of network pharmacological methods. The specific anti-inflammatory function in the initial step of PPA formation was validated in peritoneal macrophages (PMs) isolated from PPA mice on Day 3 postsurgery. The potential anti-fibrinolytic activity in the next stage of PPA formation was subsequently explored in PPA mice on Day 7 postsurgery.

**Results:**

Network pharmacology revealed 160 common targets between HXTF and PPA. Several core targets, i.e., matrix metalloproteinase 9 (MMP9), tissue-type plasminogen activator (tPA), and plasminogen activator inhibitor 1 (PAI-1), were annotated as important biological processes (extracellular matrix disassembly and the collagen catabolic process). Validation experiments revealed that HXTF could induce macrophage polarization-mediated anti-inflammatory reactions by increasing the phagocytic capacity of PMs and promoting the release of anti-inflammatory cytokines (IL-4 and IL-10). In addition, HXTF promoted fibrinogenolysis and improved fibrinolytic activity, thereby inhibiting collagen deposition and reducing adhesion development.

**Conclusion:**

The ameliorative effects of herbal HXTF on PPA formation are attributable to the induction of macrophage polarization-mediated anti-inflammatory reactions in the early stage of PPA formation and the promotion of fibrinogenolysis and fibrinolytic activity in the middle stage of PPA formation. HXTF may be a promising alternative agent for the prevention and treatment of PPA.

## 1 Introduction

Postoperative peritoneal adhesion (PPA) refers to pathologic bands connecting adjacent organs or tissues (1). The physical properties of these bands differ and include connective tissue membranes, thick fibrous bands containing blood vessels and nerves, or tight connections between abdominal organs or visceral organs (2). PPA is a consequence of abdominal irritation caused by surgery or infection (3), and abdominal surgery is the primary risk factor (4). However, the incidence of PPA cannot be completely avoided even with the widespread application of the minimally invasive approach (5–7). It has become a global health issue for humanity because of its incidence and recurrence. Approximately 90% of patients who undergo abdominal surgery develop adhesions (8). A postmortem study revealed that the incidence of abdominal surgery was as high as 43.8% in Americans over the age of 60 years (9). A recent landmark paper based on retrospective cohort studies in Scotland reported that 35% of patients were readmitted because of direct or possible adhesion-related complications (10, 11). The cost of adhesion-related readmission and abdominal reoperation places a considerable burden on individuals, families, and even society. The health costs of adhesiolysis are estimated to reach $2.1 billion per year in the United States (5), and in-hospital costs for adhesive small bowel obstruction are estimated to reach €16, 305 in the Netherlands (12).

The mechanism of PPA is complex and has not been fully elucidated. Mechanistically, when the peritoneum is stimulated by surgery, infection, or trauma, the body spontaneously becomes “self-protected”. During the first few hours to 3 days, various pathological factors, such as the aggregation of cavity macrophages in the injured serosal membrane, initiate an inflammatory reaction and trigger the initial steps and mechanisms of adhesion formation. During the subsequent healing process (5–8 days), the balance of fibrinolysis is disrupted, the production and dissolution of fibrin are imbalanced, and subsequent excessive collagen and extracellular matrix (ECM) accumulate (13, 14). The above two steps constitute the initial and middle stages of PPA formation, which are also referred to as the inflammatory and fibrinolysis responses. If the repair mechanism is delayed in this time window, the cascade of events, such as fibroblast proliferation and angiogenesis, leads to the formation of PPA (15, 16). Therefore, the development of effective agents for the inflammatory process and fibrinolytic reactions for the prevention and treatment of PPA is highly desirable.

To date, an increasing number of studies have focused mostly on antiadhesive barriers (i.e., fluid or solid gels) and pharmacological agents (e.g., aspirin and dexamethasone) to diminish the inflammatory response, activate fibrinolysis, and inhibit collagen deposition to achieve anti-PPA effects. However, different materials have different advantages and disadvantages (17). Herbal medicines possesses beneficial effects with no side effects, multiple targets, and low-cost effects (18). The *HuoXueTongFu* Formula (HXTF) originates from the classic prescription “*DaHuangMuDan* Decoction” of “Synopsis of the golden chamber” combined with clinical experience. It is mainly composed of six Chinese herbal medicines, namely, Radix Rhei Et Rhizome, Persicae Semen, Corydalis Rhizoma, Raphani Semen, Natrii Sulfas, and Carthami Flos (19). Our previous study indicated that HXTF has positive effects for preventing PPA through the SOCS/JAK2/STAT/PPAR-γ pathway (19). However, the specific mechanism by which HXTF affects PPA formation has not been fully elucidated. In the present study, we aimed to determine the critical roles of HXTF as a result of its specific anti-inflammatory and anti-fibrinolytic activities within the time window for PPA treatment.

## 2 Materials and methods

### 2.1 Reagents

The authentic standards of aloe-emodin, rhein, emodin, chrysophanol, physcion, tetrahydropalmatine, hydroxysafflor yellow A, and kaempferol used for high-performance liquid chromatography-mass spectrometry (HPLC-MS) were purchased from Yuanye Biotechnology Co., Ltd. (Shanghai, China). Fluvastatin (FS) was purchased from Novartis Pharmaceutical Co., Ltd. (Beijing, China). Methanol (Merck, Germany) and methanoic acid (Marklin, China) were used to prepare all aqueous solutions. Matrix metalloproteinase 9 (MMP9, sc-13520) was bought from Santa Cruz Biotechnology Co., Ltd. (USA). Tissue-type plasminogen activator (tPA, bs-1545R) and plasminogen activator inhibitor 1 (PAI-1, bs-1704R) were purchased from Bioss Biotechnology Co., Ltd. (Beijing, China). APC-conjugated anti-mouse CD86 (105012) was obtained from BioLegend (San Diego, USA). FITC-conjugated anti-mouse F4/80 antigen (35–4801) and PE-CY-7-conjugated anti-mouse CD206 (25–2061–82) were purchased from Thermo Fisher Technology Scientific (Massachusetts, USA). A neutral red kit (C0013) and β-actin rabbit monoclonal antibody (AF5003) were obtained from Beyotime Biotechnology Co., Ltd. (Shanghai, China). IL-4 (LA161503), IL-6 (LA160102), IL-10 (LA168604), and TNF-α (LA166601) enzyme-linked immunosorbent assay (ELISA) kits were purchased from Lapuda Biotechnology Co., Ltd. (Nanjing, China). A reverse transcription kit (H2010081) and an RNA kit (11203ES08) were obtained from Yeasen Biotechnology Co., Ltd. (Shanghai, China). A multicolor protein marker (CW2841M), SDS-PAGE gel kit (CW0022M), and eECL Western blot Kit (CW0049S) were obtained from CWBIO (Beijing, China). A Masson staining kit (DC0032) was obtained from Leagene Biotechnology Co., Ltd. (Anhui, China). Isoflurane (902–0000–522) was purchased from RWD Life Science Co., Ltd. (Shenzhen, China). Lipopolysaccharide (LPS) was purchased from Sigma Chemical (St. Louis, USA).

### 2.2 Preparation of HXTF

**Figure 1:**
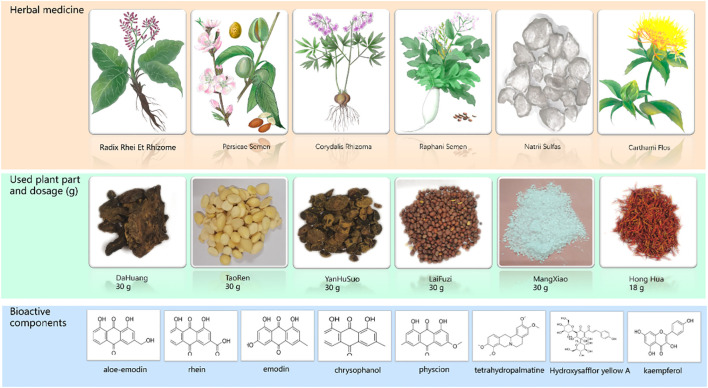
The composition of *HuoXueTongFu* Formula (HXTF) and its bioactive components (aloe-emodin, rhein, emodin, chrysophanol, physcion, tetrahydropalmatine, hydroxysafflor yellow A, and kaempferol).

All the Chinese herbal medicines (net weight: 168 g) were purchased from Jiangsu Provincial Hospital of Chinese Medicine. All the plants were identified as genuine herbs by the Processing Laboratory of Nanjing University of Chinese Medicine. The composition, dosage, and bioactive components of HXTF are shown in **Figure 1**. HXTF was prepared according to our previous studies (19, 20). Fifteen times *Dahuang*, *Taoren*, Laifuzi, and *Yanhusuo* were collected, refluxed, and extracted with 70% ethanol twice, for 2 h each time. The extracts were filtered and concentrated with a rotary evaporator until there was no alcohol flavor. *Honghua* and 20 times the amount of ultrapure water were added, and the mixture was decocted three times for 1 h each. The liquid was filtered and concentrated with a rotary evaporator until the final relative density reached 1.09–1.11. After cooling, an equal amount of anhydrous ethanol was added, the mixture was stirred evenly, and the mixture was allowed to stand for 48 h. The supernatant was removed, after which *Mangxiao* was added. Finally, the ethanol was again recovered using a rotary evaporator until no alcohol odor was detected, concentrated to a relative density of 1.15 g/mL, and stored at 4°C for use.

### 2.3 Quality evaluation of HXTF by HPLC-MS

Each standard solid portion was accurately weighed and dissolved in methanol to prepare a stock solution. A total of 40 µL of 1 mg/mL aloe-emodin, 40 μL of 0.32 mg/mL rhein, 10 μL of 1 mg/mL emodin, 40 μL of 0.32 mg/mL chrysophanol, 80 μL of 0.16 mg/mL physcion, 5 μL of 2 mg/mL tetrahydropalmatine, 20 μL of 1 mg/mL hydroxysafflor yellow A, and 10 μL of 1 mg/mL kaempferol were collected and diluted to 10 mL, and 1.0 mL of HXTF was diluted to 10 mL, which was used for the HPLC-MS system. The operation was performed using a TSQ Quantis mass spectrometry system (Thermo Fisher Scientific, USA) with an ACQUITY UPLC^®^BEH C18 column (2.1 × 100 mm, 1.7 μm). Mobile phase A and mobile phase B were methanol and 0.1% methanoic acid, respectively. The gradient was as follows: 0–5 min: 10%–15% B; 5–12 min: 15%–55% B; 12–17 min: 55%–75% B; 17–25 min: 75%–90% B; 25–28 min: 90%–95% B; 28–30 min: 95% B; 30–31 min: 95%–10% B; and 31–34 min: 10% B. The flow rate was 0.3 mL/min, injection volume was 5 μL, and the column temperature was 30°C.

### 2.4 Network pharmacology of HXTF and PPA

#### 2.4.1 Screening of potential targets of HXTF and PPA

The PubChem ID and 2D chemical structure of HXTF active components (aloe-emodin, rhein, emodin, chrysophanol, physcion, tetrahydropalmatine, hydroxysafflor yellow A, and kaempferol) in the fingerprint were downloaded from the PubMed platform (https://pubmed.ncbi.nlm.nih.gov/) and PubChem database (https://pubchem.ncbi.nlm.nih.gov/). The target information was obtained from the Swiss Target Prediction Database (http://www.swisstargetprediction.ch/SWISS), the Comparative Toxicogenomics Database (http://ctdbase.org/), and the Traditional Chinese Medicine Systems Pharmacology Database and Analysis Platform (http://lsp.nwu.edu.cn/index.php). The related target genes were extracted from the UniProt database (https://www.uniprot.org), which is regarded as a unified standard (21, 22). The targets related to PPA were acquired from the GeneCards database (https://www.genecards.org) and the OMIM database (https://omim.org) by searching for “postoperative peritoneal adhesion” or “abdominal adhesion” as keywords. We searched for disease-related targets of PPA, screened genes with a score ≥ 7.36 (7.36 as the median score), and established disease target datasets.

#### 2.4.2 Construction of the HXTF–PPA common target network

The common targets of HXTF and PPA were obtained by analyzing the related targets of HXTF and PPA. A Venn diagram was drawn using the bioinformatics platform (http://www.bioinformatics.com.cn/). To reflect the complex relationships among HXTF and PPA, an HXTF–PPA common target network was established visually on the basis of the aforementioned datasets using Cytoscape v3.8.2 (http://www.cytoscape.org/).

#### 2.4.3 Construction of a protein-protein interaction (PPI) network and screening of core targets

In this study, the Search Tool for the Retrieval of Interacting Genes (STRING) database (https://string-db.org/) was used to construct possible protein-protein interactions (PPIs) by uploading the common targets related to HXTF and PPA. The species were limited to “*Homo sapiens*” with a confidence score > 0.400. Then, Cytoscape was used to construct the PPI core target networks. A network analyzer in Cytoscape was used to analyze the topological parameters of the mean and maximum degrees of freedom in the PPI network. The core targets were screened according to the value of the degrees. The larger the value, the more likely the target is to become the core target of HXTF in the treatment of PPA.

#### 2.4.4 Gene Ontology and pathway analysis

Gene Ontology (GO) defines the concepts of gene function and the interrelationships among the functions of different genes, including molecular function (MF), cellular component (CC), and biological process (BP). KEGG pathway analysis involves a collection of databases describing biological pathways, genomes, drugs, and diseases. It can be used to study the biological effects and multidimensional pharmacological mechanisms of the core targets at the pathway level. GO annotation and pathway analysis were conducted using the DAVID online tool (https://david.ncifcrf.gov/). A *p* < 0.01 was taken as the critical value of significant function and pathway, and the organism was selected as “*Homo sapiens*”.

### 2.5 Experimental verification

#### 2.5.1 Experimental animals and preparation

Eighty male C57BL/6J mice aged 6–8 weeks and weighing approximately 20 g were purchased from Hangzhou Medical College (Hangzhou, China) and raised at the animal experimental center of Nanjing University of Chinese Medicine. The mice were kept in a room with a suitable environment (12-h light/dark cycle, room temperature of approximately 25°C, and relative humidity of approximately 50%) and were allowed to eat and drink freely. All experiments were conducted following the current institutional ethics for animal care and were authorized by the Ethical Committee of Nanjing University of Chinese Medicine (ethics No. 202009A036).

#### 2.5.2 Experimental design and surgical procedure

The mice were fasted for 12 h and allowed access to water the night before the surgery. The mice were randomly divided into four groups: (1) sham group (*n* = 20); (2) PPA group (*n* = 20); (3) HXTF+PPA group (0.85 g/100 g, *n* = 20); and (4) fluvastatin + PPA group (FS+PPA, 0.5 mg/100 g, *n* = 20). FS, an HMG-CoA reductase inhibitor with antioxidant, anti-inflammatory, and pro-fibrinolytic effects, is a potent fibrinolytic modulator under both normal and inflammatory conditions and plays a role in the formation of postoperative adhesion (23, 24). Thus, FS was used as a positive control. Ten mice in each group treated for 3 days were used for peritoneal macrophage (PM) collection, while the other 10 mice in each group treated for 7 days were used for rodent experiments. The PPA model was prepared as previously described (19, 25). In general, the mice underwent skin preparation after being anesthetized with 1.5% isoflurane. The mice were fixed in the supine position, and their abdomen was disinfected three times with iodophor. A longitudinal incision of approximately 2 cm was made in the anterior midline of the lower abdomen. The cecum was removed on the wet gauze and rubbed repeatedly with sterile gauze until prickly bleeding appeared on the surface, resulting in a wound of approximately 1.5 cm × 1.5 cm. The procedure lasted for approximately 3 min. The cecum was then gently returned to the abdomen, and the abdominal wall was sutured. The mice in the sham group were exposed to air for 3 min without friction and were administered normal saline once a day within 3 or 7 days after the surgery. The mice in the HXTF group were gavaged with herbal HXTF once a day within 3 or 7 days after the operation, and the mice in the FS group were gavaged with FS once a day within 3 or 7 days after the surgery.

#### 2.5.3 Peritoneal resident macrophage isolation

Macrophages are key cells in tissue homeostasis and inflammation, which can polarize into classical M1 and alternative M2 phenotypes. PMs were isolated and cultured as previously described (26). Briefly, 10 mice in each group were sacrificed via cervical dislocation 2 h after intragastric administration on Day 3 postsurgery, and 4–5 mL of preheated DMEM medium was intraperitoneally injected. After the abdomen was kneaded for 10 min, 5 mL of PBS with 5 mM EDTA was injected to wash the peritoneal cavity. The cells were harvested gently, counted with a cell counter, and cultured in an incubator for 24 h until full wall attachment was achieved. After removing and counting the nonadherent cells, PMs were obtained and incubated for another 48 h. The number of PMs was equal to the total number of cells minus the number of nonadherent cells (27).

#### 2.5.4 Neutral red assay of the phagocytic capacity of PMs

The PMs isolated from different groups were plated at 2×10^4^ per well in 96-well plates for 24 h. The cells were treated with vehicle or LPS (1 μg/mL) for another 24 h. After incubation with the neutral red kit according to the instructions, the OD values were detected at a wavelength of 690 nm using an enzymatic analyzer (Bio-Rad, USA).

#### 2.5.5 Flow cytometric analysis of M1 and M2 polarization

PMs were incubated with FITC-conjugated anti-mouse F4/80 (0.5 μg/10^6^ cells) and APC-conjugated anti-mouse CD86 (0.25 μg/10^6^ cells) at 4°C for 40 min, stained with 100 μL of IC fixation buffer at room temperature for 30 min, and incubated with PE-CY-7-conjugated anti-mouse CD206 (0.25 μg/10^6^ cells) at room temperature for another 60 min. After being cleaned with permeabilization buffer three times, PMs were detected via flow cytometry (Amnis, Millipore, USA) and analyzed using IDEAS software.

#### 2.5.6 ELISA of M1 phenotype-related proinflammatory cytokines and M2 phenotype-related anti-inflammatory factors

The supernatant of the PMs was centrifuged at 3,000 rpm and collected in a new tube. ELISA was performed according to the manufacturer’s instructions. Briefly, samples or standards were incubated with biotinylated anti-IL-4, anti-IL-6, anti-IL-10, and anti-TNF-α reagents within 96-well plates at 37°C for 1.5 h. Following a washing step with buffer, streptavidin-HRP was added to each well and incubated at 37°C for 0.5 h. After washing with buffer, TMB and stop solution were added to react with the enzyme–antibody–target complex to produce a measurable signal. The intensity of this signal was directly measured at 450 nm using a microplate reader.

#### 2.5.7 Macroscopic evaluation of the adhesion score

One hour after oral administration on Day 7 postsurgery, the remaining 10 mice in each group were sacrificed. The abdominal cavity was opened with a U-shaped incision. Adhesion of the abdominal cavity was observed and evaluated according to Nair’s classification standard (**Table 1**) by two technicians who were blinded to the experimental conditions.

**Table 1:** Nair’s classification standard (28).

Grade	Description	Score
0	No adhesions	0
I	A single cord of adhesion between viscera or between viscera and abdominal wall	1
II	Double bands between viscera or between viscera and abdominal wall	2
III	More than two bands between viscera or between viscera and abdominal wall	3
IV	The viscera adhered directly to the abdominal wall regardless of the number and degree of adhesion	4

#### 2.5.8 Histopathological assessments of adhesive tissue

The cecum tissue was fixed in 4% paraformaldehyde for 24 h, embedded in paraffin, and sliced into 4-μm-thick pathological sections. The sections were subjected to Masson’s trichrome staining.

#### 2.5.9 Western blot analysis of fibrinogenolytic and fibrinolytic indicators

The proteins of cecum tissue were prepared, quantified, and transferred onto a PVDF membrane by 10% sodium dodecyl sulfate-polyacrylamide gel electrophoresis. The membranes were blocked with 5% milk for 1 h and incubated with primary antibody at 4°C overnight. Then, the membranes were stained with the secondary antibody for another 2 h at room temperature. Finally, the protein expression levels were detected using a chemiluminescent gel imaging system (Bio-Rad, USA), and the gray values were analyzed via ImageJ.

#### 2.5.10 Real-time RCR of fibrinogenolytic and fibrinolytic indicators

Total RNA of cecum tissue was extracted with TRIzol reagent (Invitrogen, USA) and concentrated with a Nanodrop 2000. cDNA was synthesized according to the instructions. All the results were analyzed using the double-delta method (2^−ΔΔCt^). The primer information is shown in **Table 2**.

**Table 2:** Primers for real-time PCR.

Name	Oligo	Primer sequence (5′–3′)
CD86	Forward primer	TGTTTCCGTGGAGACGCAAG
	Reverse primer	TTGAGCCTTTGTAAATGGGCA
CD206	Forward primer	CTCTGTTCAGCTATTGGACGC
	Reverse primer	CGGAATTTCTGGGATTCAGCTTC
MMP9	Forward primer	CTGGACAGCCAGACACTAAAG
	Reverse primer	CTCGCGGCAAGTCTTCAGAG
tPA	Forward primer	TGACCAGGGAATACATGGGAG
	Reverse primer	CTGAGTGGCATTGTACCAGGC
PAI-1	Forward primer	TTCAGCCCTTGCTTGCCTC
	Reverse primer	ACACTTTTACTCCGAAGTCGGT
GAPDH	Forward primer	CTGGAGAAACCTGCCAAGTATG
	Reverse primer	GGTGGAAGAATGGGAGTTGCT

### 2.6 Statistical analysis

All the data were analyzed via SPSS 25.0 (Chicago, IL, USA), and all the results are expressed as the means ± standard deviations. One-way analysis of variance (ANOVA) and the least significant difference (LSD) test were used to compare normally distributed data. The Kruskal-Wallis test was used to analyze nonnormally distributed data. Fisher’s exact test was applied to process the count data. *p* < 0.05 was considered statistically significant.

## 3 Results

### 3.1 Chromatogram peaks of HXTF by HPLC-MS

**Figure 2:**
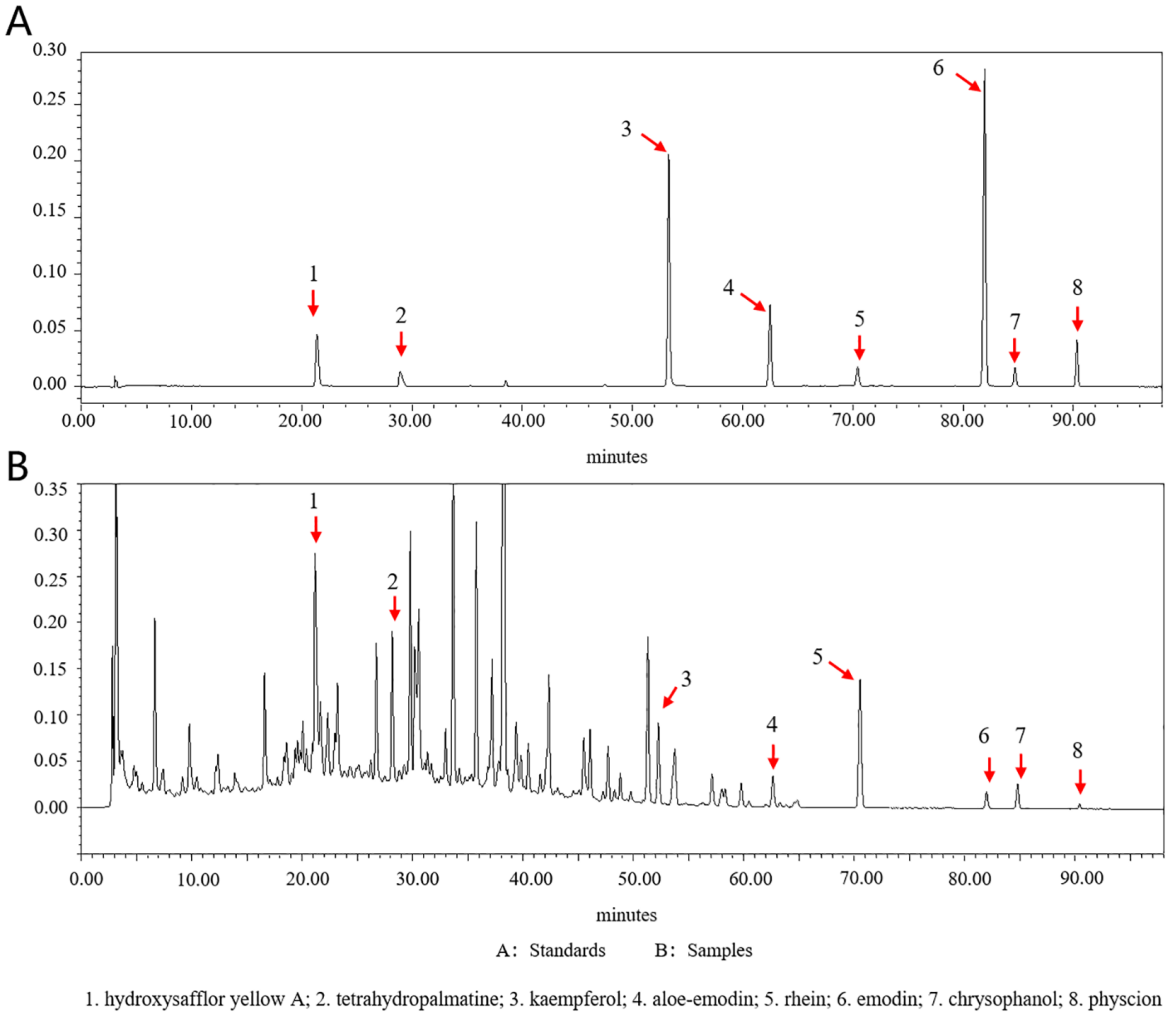
Chromatograms of eight standards and eight analytes in HXTF. **(A)** Standards. **(B)** Samples. (1) hydroxysafflor yellow A; (2) tetrahydropalmatine; (3) kaempferol; (4) aloe-emodin; (5) rhein; (6) emodin; (7) chrysophanol; (8) physcion.

Peaks of eight analytes in HXTF and calibration standards (aloe-emodin, rhein, emodin, chrysophanol, physcion, tetrahydropalmatine, hydroxysafflor yellow A, and kaempferol) were identified by comparison of the retention times (RTSs) and MS spectra (**Table 3**, **Figure 2**).

**Table 3:** Mass spectrometric parameters of eight components.

Compounds	Polarity	Precursor (m/z)	Product (m/z)	Collision energy (V)	Retention time (RTS)
Aloe-emodin	Negative	269.25	240	20.92	16.00
Rhein	Negative	283.35	239	18.45	17.70
Emodin	Negative	269.30	225	25.62	19.60
Chrysophanol	Negative	253.25	225	27.14	20.18
Physcion	Negative	284.30	240	25.77	21.80
Tetrahydropalmatine	Positive	356.20	192	27.29	10.30
Hydroxysafflor yellow A	Positive	613.44	318	15.23	8.17
Kaempferol	Negative	285.30	239	27.57	14.83

### 3.2 Mechanistic effect of HXTF on PPA treatment based on network pharmacology

**Figure 3:**
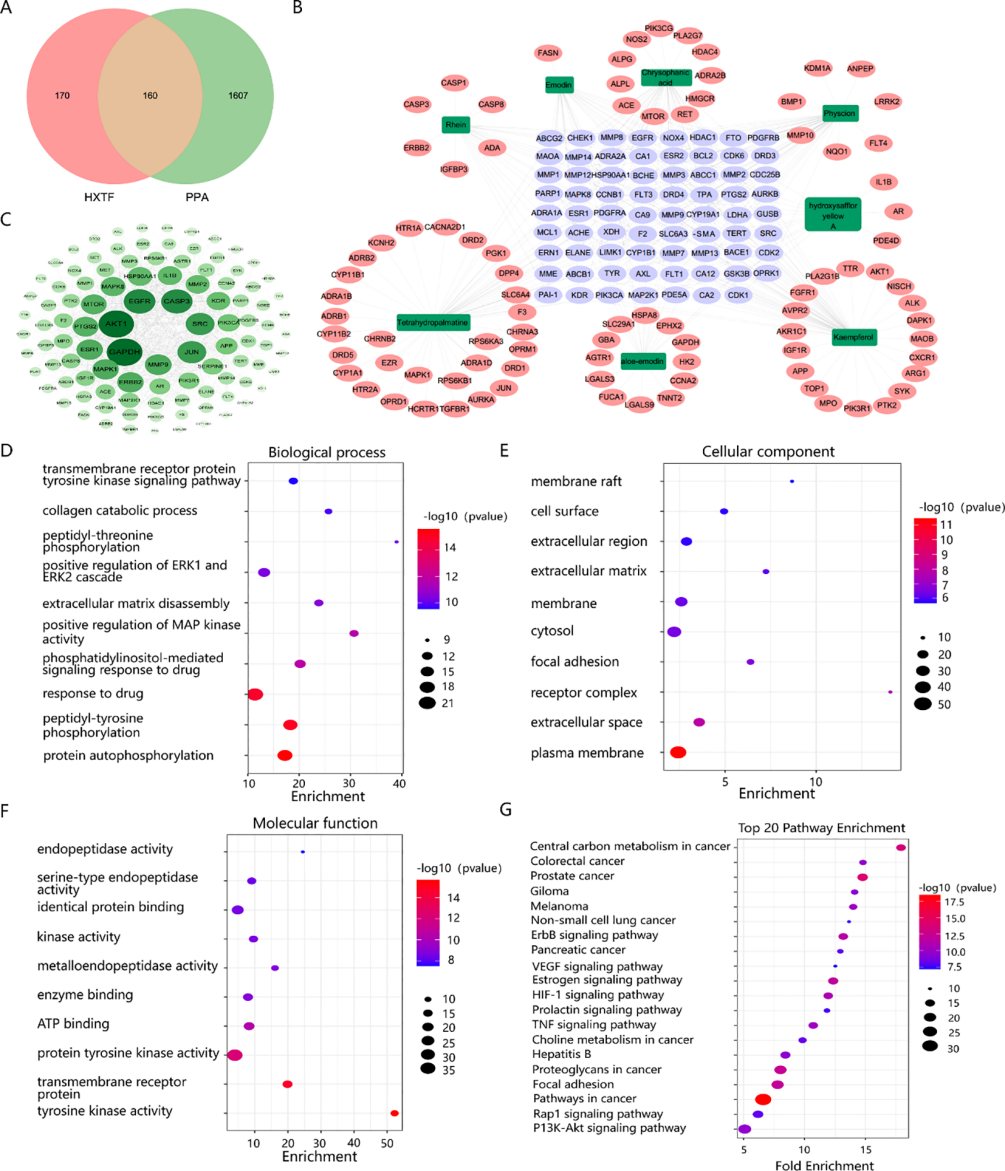
Network pharmacology analysis of the effects of HXTF on PPA treatment. **(A)** Venn diagram showing the overlapping genes among the active components of HXTF and the targets of PPA. **(B)** Network construction of HXTF and PPA common targets by using the Cytoscape tool. The green nodes represent the eight main components of HXTF, the purple nodes represent their common target, and the pink nodes represent their single target. Edges represent the interactions between nodes. **(C)** PPI network construction and core target identification by using the Cytoscape tool. The color and depth of size are proportional to the degree value. **(D)** The top 10 GO annotations in BP by using the DAVID online tool. **(E)** The top 10 GO annotations in CC by using the DAVID online tool. **(F)** The top 10 GO annotations in MF by using DAVID online tool. **(G)** The top 20 enriched KEGG pathways by using the DAVID online tool. HXTF, *HuoXueTongFu* Formula; PPA, postoperative peritoneal adhesion.

A total of 330 targets of eight bioactive components (hydroxysafflor yellow A, tetrahydropalmatine, kaempferol, aloe-emodin, rhein, emodin, chrysophanol, and physcion) of HXTF were predicted, and 1,767 PPA-related targets were identified. An analysis of the potential targets of HXTF and PPA revealed 160 overlapping targets, as shown in **Figure 3A**, suggesting a close relationship with PPA treatment.

To understand the synergistic effects of multiple components and multiple targets of HXTF on PPA treatment, a network topology was constructed, which contained 157 nodes and 1,847 edges, as presented in **Figure 3B**. PPI network construction and core target identification could help us identify the underlying molecular mechanism involved. According to the topological properties of the degree of network nodes, the greater the degree value, the greater were the color and depth of size, as shown in **Figure 3C**. Among the top 20 genes, tPA (degree = 71), MMP9 (degree = 66), and PAI-1 (degree = 46), which could serve as kernel targets of HXTF in PPA treatment, were identified.

GO annotation was performed to identify potential biological functions. As a result, 358 BPs, 60 CCs, and 97 MFs were obtained (*p* < 0.01), and the top GO terms are presented in **Figures 3D–F**. We found that HXTF could regulate ECM disassembly and collagen catabolic processes during the treatment of PPA. Several core targets, i.e., MMP9, tPA, and PAI-1, were annotated in the above biological processes. In addition, 82 enriched pathways were identified, suggesting that HXTF treatment of PPA was closely related to the HIF-1 and TNF signaling pathways. The top 20 pathways are shown in **Figure 3G**. Together, the network pharmacology findings indicated that MMP9, tPA, and PAI-1 were the core targets of HXTF in PPA treatment via their regulation of ECM disassembly and collagen catabolic processes, which were closely associated with the HIF-1 and TNF signaling pathways.

### 3.3 HXTF could increase the phagocytic capacity of PMs

**Figure 4:**
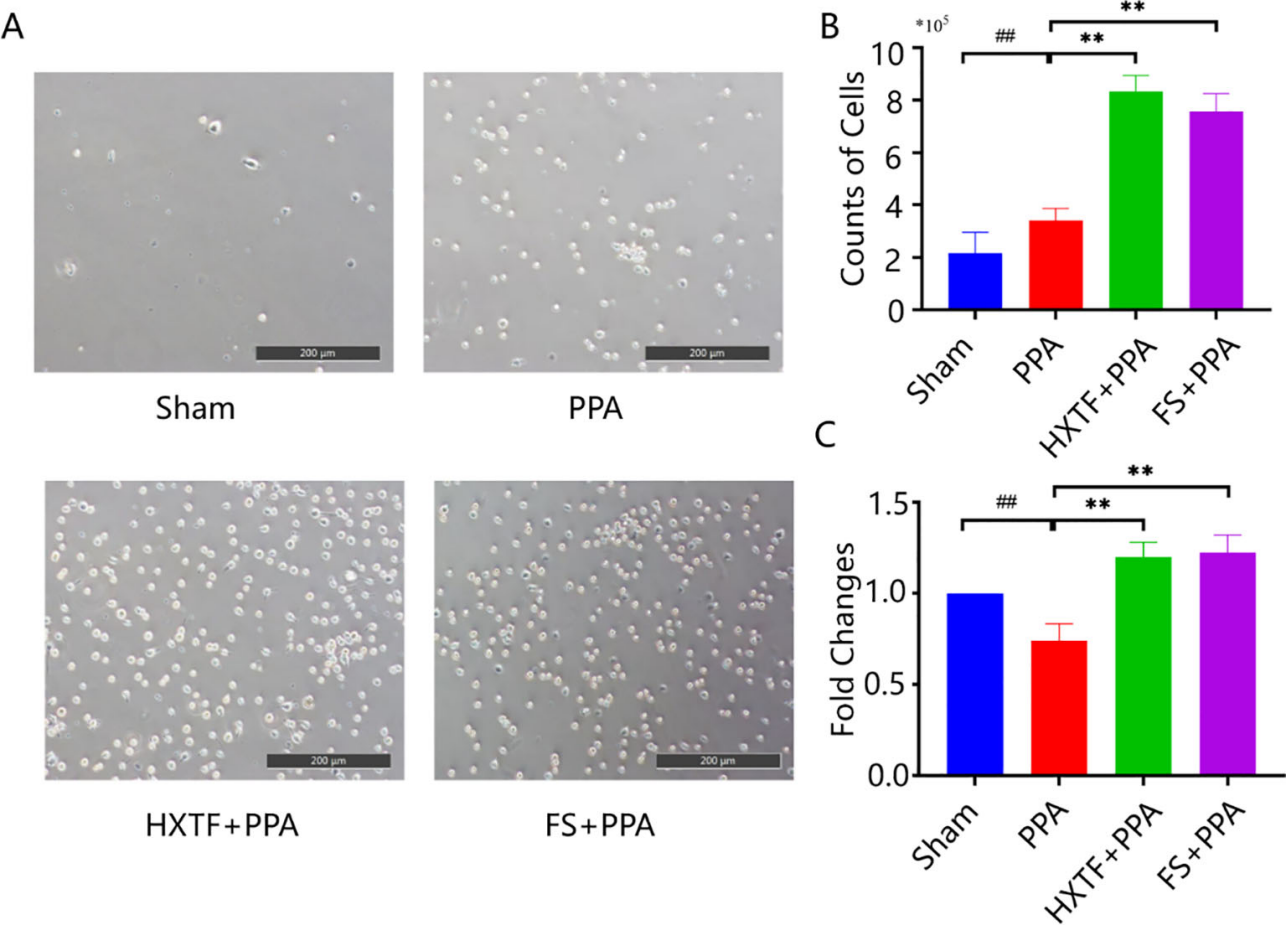
HXTF enhanced the phagocytic capacity of PMs. **(A)** Random views of PMs from the abdominal cavity of each group at 200× magnification. **(B)** The number of PMs in each group. Compared with the sham group, ^##^ *p* < 0.01. Compared with the PPA group, ^**^ *p* < 0.01. **(C)** The phagocytic capacity of PMs in each group. Compared with the sham group, ^##^ *p* < 0.01. Compared with the PPA group, ^**^ *p* < 0.01. HXTF, *HuoXueTongFu* Formula; PPA, postoperative peritoneal adhesion; PMs, peritoneal macrophages; FS, fluvastatin.

Macrophages are plastic cells that eliminate apoptotic or necrotic cells through specific phagocytic processes. The function of macrophages depends on their polarization activity, including the M1 or M2 phenotype (29, 30). PMs initially appeared circular or ovoid with high wall-adherent speed. After being cultured *in vitro*, the cells had pseudopodia and small protrusions and some exhibited a shuttle shape, as shown in **Figure 4A**. The results of cell counting revealed that the numbers of PMs obtained from the abdominal cavity in the sham, PPA, HXTF, and FS groups were (2.15 ± 0.80)×10^5^, (3.40 ± 0.47)×10^5^, (8.33 ± 0.61)×10^5^, and (7.57 ± 0.69)×10^5^, respectively, as shown in **Figure 4B**. There was a significant difference in the phagocytic capacity of PMs in the HXTF group compared with the PPA group (*p* < 0.01), as presented in **Figure 4C**. Taken together, these results suggested that HXTF could increase the phagocytic capacity of PMs.

### 3.4 HXTF could induce macrophage polarization-mediated anti-inflammatory reactions

**Figure 5:**
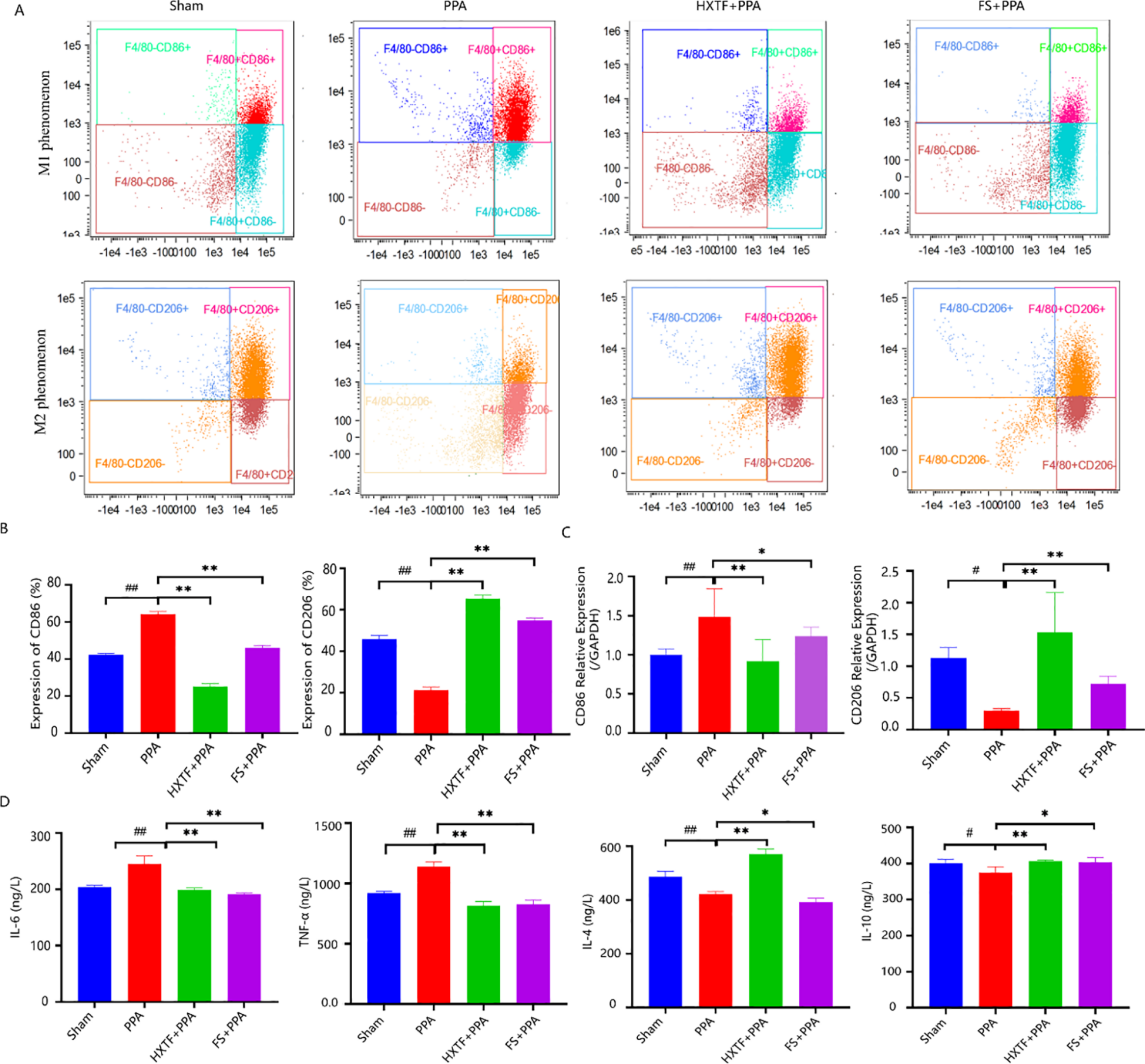
HXTF induced macrophage polarization-mediated anti-inflammatory reactions. **(A, B)** Flow cytometry analysis of the expression of the M1/M2 markers CD86 and CD206 in each group. Compared with the sham group, ^##^ *p* < 0.01. Compared with the PPA group, ^**^ *p* < 0.01. **(C)** Real-time PCR assay of the mRNA levels of CD86 and CD206 in each group. Compared with the sham group, ^#^ *p* < 0.05, ^##^ *p* < 0.01. Compared with the PPA group, ^*^ *p* < 0.05, ^**^ *p* < 0.01. **(D)** ELISA analysis of M1 phenotype-related proinflammatory cytokines (IL-6 and TNF-α) and M2 phenotype-related anti-inflammatory factors (IL-4 and IL-10) in each group. Compared with the sham group, ^#^ *p* < 0.05, ^##^ *p* < 0.01. Compared with the PPA group, ^*^ *p* < 0.05, ^**^ *p* < 0.01. HXTF, *HuoXueTongFu* Formula; PPA, postoperative peritoneal adhesion; FS, fluvastatin.

F4/80, CD86, and CD206 are typical markers of macrophages, M1 macrophages, and M2 macrophages, respectively. To understand the M1/M2 polarization trend of PMs, the above typical markers were detected via flow cytometry. The results revealed that the levels of CD86 and CD206 in the PPA group were 64.1% ± 1.50% and 21.23% ± 1.46%, respectively. After treatment with HXTF, the expression of CD206 increased to 65.37% ± 1.70%, and the expression of CD86 decreased to 25.13% ± 1.55%, as presented in **Figures 5A, B**. These results were consistent with those of the qRT-PCR analysis (**Figure 5C**). The balance of M1 macrophages (M1) and M2 macrophages (M2) plays key roles in macrophage-mediated inflammatory reactions (31). M1 phenotype-related proinflammatory cytokines (IL-6 and TNF-α) and M2 phenotype-related anti-inflammatory factors (IL-4 and IL-10) were detected via ELISA. Compared with those in the sham group, the levels of IL-6 and TNF-α in the PPA group were elevated, and the levels of IL-4 and IL-10 were decreased. After treatment with HXTF, the levels of IL-6 and TNF-α were significantly decreased, and the levels of IL-4 and IL-10 were markedly increased, as shown in **Figure 5D**. Collectively, these data demonstrated that HXTF could induce macrophage polarization toward the M2 phenotype and promote the release of anti-inflammatory cytokines, thereby regulating macrophage-mediated inflammatory reactions.

### 3.5 HXTF could reduce the adhesion score and inhibit collagen deposition

**Figure 6:**
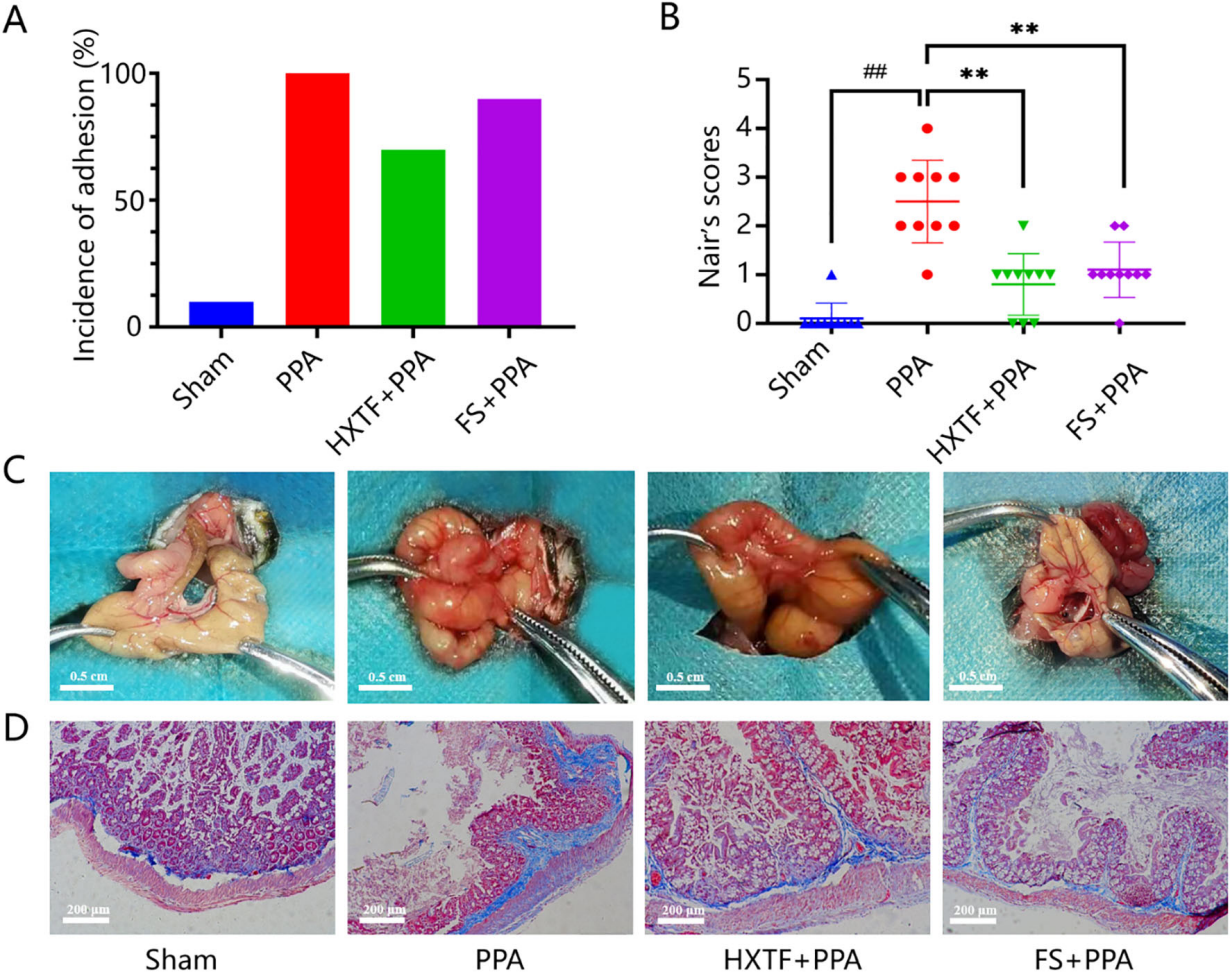
HXTF reduced the adhesion score and inhibited collagen deposition. **(A)** Adhesion rates in each group. **(B)** Nair scores for each group. Compared with the sham group, ^##^ *p* < 0.01. Compared with the PPA group, ^**^ *p* < 0.01. **(C)** Representative images of adhesion formation in each group (*n* = 10). **(D)** Masson staining of each group at 200× magnification. HXTF, *HuoXueTongFu* Formula; PPA, postoperative peritoneal adhesion; FS, fluvastatin.

There were no mouse deaths or postoperative complications during model preparation or subsequent intervention. There was no significant difference in the weights of the mice in the four groups. The incidence of adhesion and Nair scores were as follows: PPA > FS > HXTF > Sham, as shown in **Figures 6A, B**. Compared with the sham group, the PPA group presented severe peritoneal adhesion with the highest adhesion rate and score. Macroscopically, the adhesive bands of the mice in the PPA group were more extensive and thicker, and some of the cecum directly adhered to the abdominal wall. After treatment with HXTF, the adhesive bands were looser and easier to peel off, resulting in a significantly reduced adhesion score. Representative images of adhesion formation are shown in **Figure 6C**. In addition, there were massive compactly arranged collagen fibers and fiber bands connected with the serosal layer in the PPA group. Only a few loosely arranged collagen fibers with minor bands were found in the HXTF group (**Figure 6D**). Taken together, the results revealed that HXTF could reduce the adhesion score and inhibit collagen deposition.

### 3.6 HXTF promoted fibrinogenolysis and improved fibrinolytic activity

**Figure 7:**
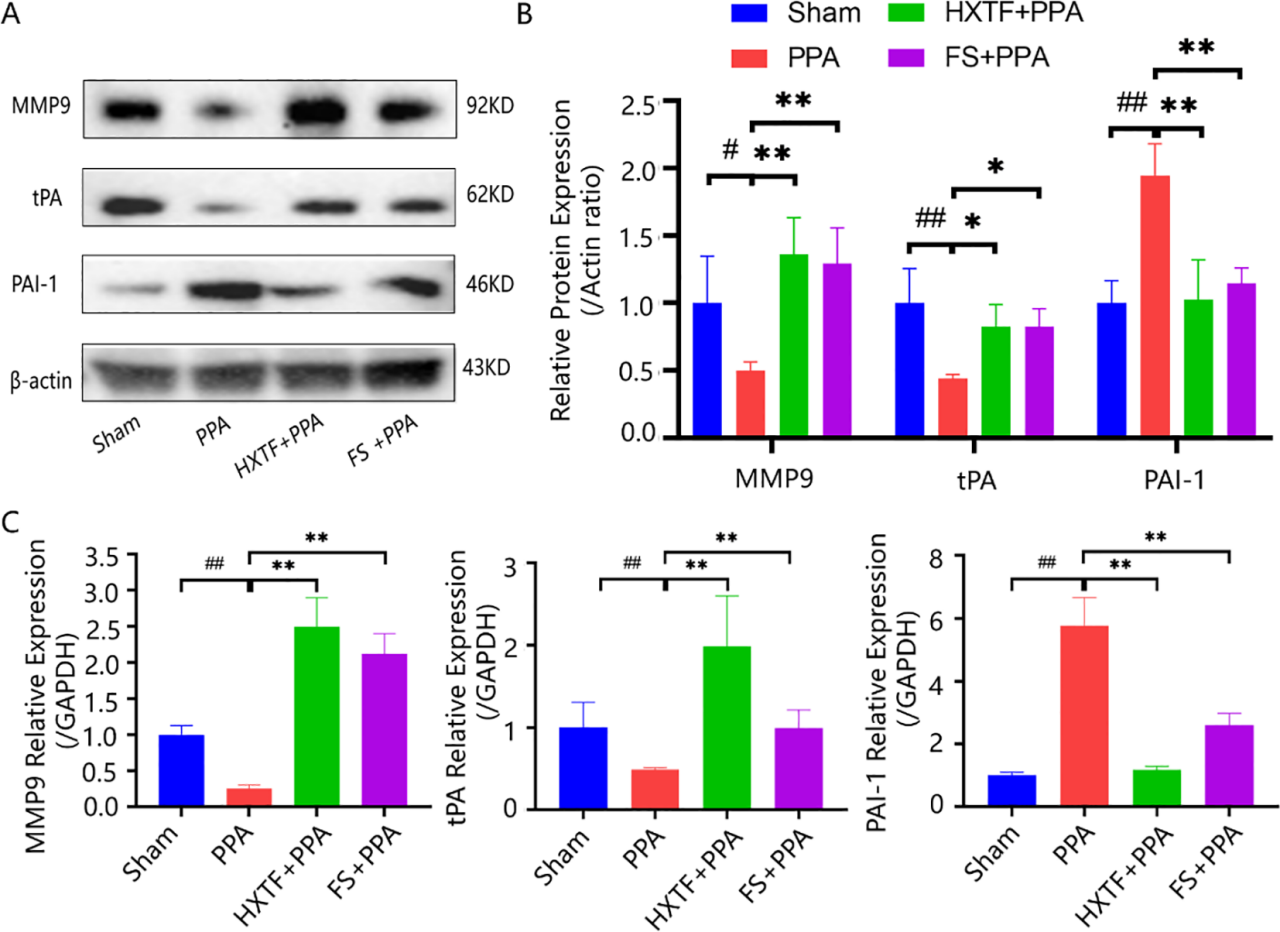
HXTF promoted fibrinogenolysis and improved fibrinolytic activity. **(A, B)** Western blot analysis of MMP9, tPA, and PAI-1 protein expression in PPA mice in each group. Compared with the sham group, ^#^ *p* < 0.05, ^##^ *p* < 0.01. Compared with the PPA group, ^*^ *p* < 0.05, ^**^ *p* < 0.01. **(C)** Real-time PCR assay of the MMP9, tPA, and PAI-1 levels in PPA mice in each group. Compared with the sham group, ^##^ *p* < 0.01. Compared with the PPA group, ^**^ *p* < 0.01. HXTF, *HuoXueTongFu* Formula; PPA, postoperative peritoneal adhesion; FS, fluvastatin.

The above GO enrichment analysis indicated that ECM disassembly and the collagen catabolic process were the major biological processes affected by HXTF in the treatment of PPA. An imbalance in collagen production and degradation and abnormal fibrinolytic activity are important reasons for adhesion formation (32). tPA and PAI-1 are pivotal regulators of fibrin deposition in the fibrinolytic system (33). MMP9 is a key enzyme with strong collagen hydrolysis activities (3). To explore the underlying mechanism of the effects of HXTF on PPA, three related indicators (MMP9, tPA, and PAI-1) related to the process of collagen and ECM deposition were analyzed at the molecular level. The results of the Western blot analysis demonstrated that the protein expression levels of MMP9 and tPA in the adhesion tissues were markedly lower in the PPA group than in the sham group. After HXTF treatment, the protein levels were significantly increased. Compared with that in the sham group, the protein expression of PAI-1 was elevated in the PPA group. After treatment with HXTF, the protein expression decreased (**Figures 7A, B**). The results of qRT-PCR were consistent with those of the Western blot analysis, as presented in **Figure 7C**. Taken together, these findings suggested that HXTF could promote fibrinogenolysis and improve fibrinolytic activity.

## 4 Discussion

PPA is one of the most severe complications after surgery, which is a major cause of bowel obstruction and infertility (34). With improvements in surgical methods and the application of mechanical materials or antiadhesive adjuvants, the formation of PPA has improved to a certain extent, mostly in rodent models. However, the high incidence rate (8, 9) and high medical costs (5, 12) are still major problems worldwide. The formation of PPA is a strictly timed process that starts with peritoneal injury, is initiated by the inflammatory response, and erupts during local repair. Acute inflammation reactions occur within a few minutes with increased vasopermeability. Neutrophils and macrophages aggregate at injured lesion sites to form fibrin networks. Fibrinogen exudation and fibrin deposition act as cell scaffolds in 3–7 days, causing excessive ECM deposition and vascularization, eventually leading to the formation of dense adhesions within 7–14 days (35, 36). Another study reported that the time for bridging adhesion is approximately 3 to 5 days (37). Therefore, this time window is crucial for adhesion development or peritoneal healing. It is very important to identify effective approaches and agents to reduce PPA formation within the time-to-treatment window. Fortunately, as a classic herbal formulation, HXTF can significantly reduce pathological damage to the peritoneum in model rats (19). However, the exact effects and mechanism of action of HXTF on PPA formation within this time window have not been fully elucidated. In the present study, we integrated various information from publicly available databases to predict interactions between HXTF and its potential targets in PPA. The findings were validated by cell and animal studies.

Recent studies have reported the protective and pathological contributions of PMs to PPA formation (38–40). Macrophage phagocytosis is a pivotal step in the body’s defense (41). Macrophages can alleviate inflammation not only by endocytosis but also by altering M1/M2 polarization (42). M2 macrophages have anti-inflammatory properties. The phagocytic capacity of M2 phenotype macrophages is better than that of M1 phenotype macrophages (26). Their powerful phagocytic capacity can not only remove cell debris and apoptotic cells but also promote peritoneum repair and wound healing (43). Given the role of macrophages in the occurrence and development of PPA, PMs isolated from PPA mice in the early stage (Day 3 postsurgery) were detected with a neutral red kit. The phagocytic ability of PMs treated with HXTF was the strongest, indicating that HXTF could effectively enhance the phagocytic function of macrophages and reduce the damage caused by excessive inflammation. Moreover, we found that PMs treated with HXTF presented an obvious M2 polarization trend, as evidenced by flow cytometry and real-time PCR. In the HXTF group, the levels of M2 phenotype-related anti-inflammatory factors (IL-4 and IL-10) were increased, and the levels of M1 phenotype-related proinflammatory cytokines (IL-6 and TNF-α) were decreased. These results are consistent with our previous findings in murine macrophage RAW264.7 cells (19). The present data provide evidence that HXTF promotes macrophage polarization-mediated anti-inflammatory responses in the initial stage of PPA development.

Notably, a series of network pharmacological methods were applied to explore the molecular mechanism of HXTF in PPA formation from a systemic perspective. By constructing PPI and “component-target-disease” networks, 160 common targets involved in the anti-PPA effect of HXTF were identified. Several core targets (i.e., tPA, MMP9, and PAI-1) with relatively high degrees were identified. The key targets of HXTF for PPA prevention and treatment were enriched in ECM disassembly and the collagen catabolic process, as well as the HIF-1 and TNF signaling pathways. The findings also revealed the potential of HXTF as a promising agent for PPA prevention and treatment.

Importantly, the above pharmacological results prompted us to further explore the pathogenesis of PPA in the middle stage after peritoneal injury (Day 7 postsurgery) using animal experiments. Inflammation rapidly occurs after peritoneal injury, followed by the formation of fibrous exudates and fibrin. The outcomes of collagen deposition and degradation within 7 days after the surgery determine normal peritoneal healing or adhesion formation (44). Inactive plasminogen is transformed into active plasmin by tPA, a major plasminogen activator that is involved in the degradation of fibrin and the ECM, thereby greatly reducing excessive deposition. PAI-1, as the major regulator of the plasminogen system, not only inhibits the activity of plasminogen but also is the most effective inhibitor of tPA (45). The imbalance between the increased levels of PAI-1 and the decreased levels of tPA is considered to be the main reason for PPA development (46). As essential enzymes for ECM cleavage, MMPs can not only mediate the neutrophil response to inflammation, improve inflammation, and promote healing, but also promote ECM deposition by degrading the common TNF-α, producing degradation products, and promoting fibrinolytic balance (47). MMP9, a member of the MMP family, plays an important role in ECM reconstruction by degrading collagen, cytokines, growth factors, and their receptors (48). It can not only regulate the dynamic balance of the ECM but also hydrolyze collagen proteins to delay the progression of peritoneal fibrosis (49). Combined with the histopathological results and adhesion scores, our results collectively revealed the ameliorative effects of HXTF in reducing collagen deposition and promoting fibrinolysis in the second stage of PPA progression at the systemic and molecular levels.

However, our study has several limitations. First, a quantitative analysis of the major bioactive components of HXTF is lacking. It is unclear which ingredients play primary protective roles in PPA, even though one study has indicated that emodin can significantly reduce abdominal adhesion formation in a rat model (50). Second, the herbal formula has multiple components, multiple targets, and multiple pathways to exert its pharmacological effects. Further efforts are needed to explore the key pathways, i.e., the HIF-1 and TNF signaling pathways, to elucidate the synergistic effects of HXTF in the prevention and treatment of PPA. Third, the potential mechanisms of HXTF in the subsequent steps of PPA development require exploration in our future research.

## 5 Conclusion

**Figure 8:**
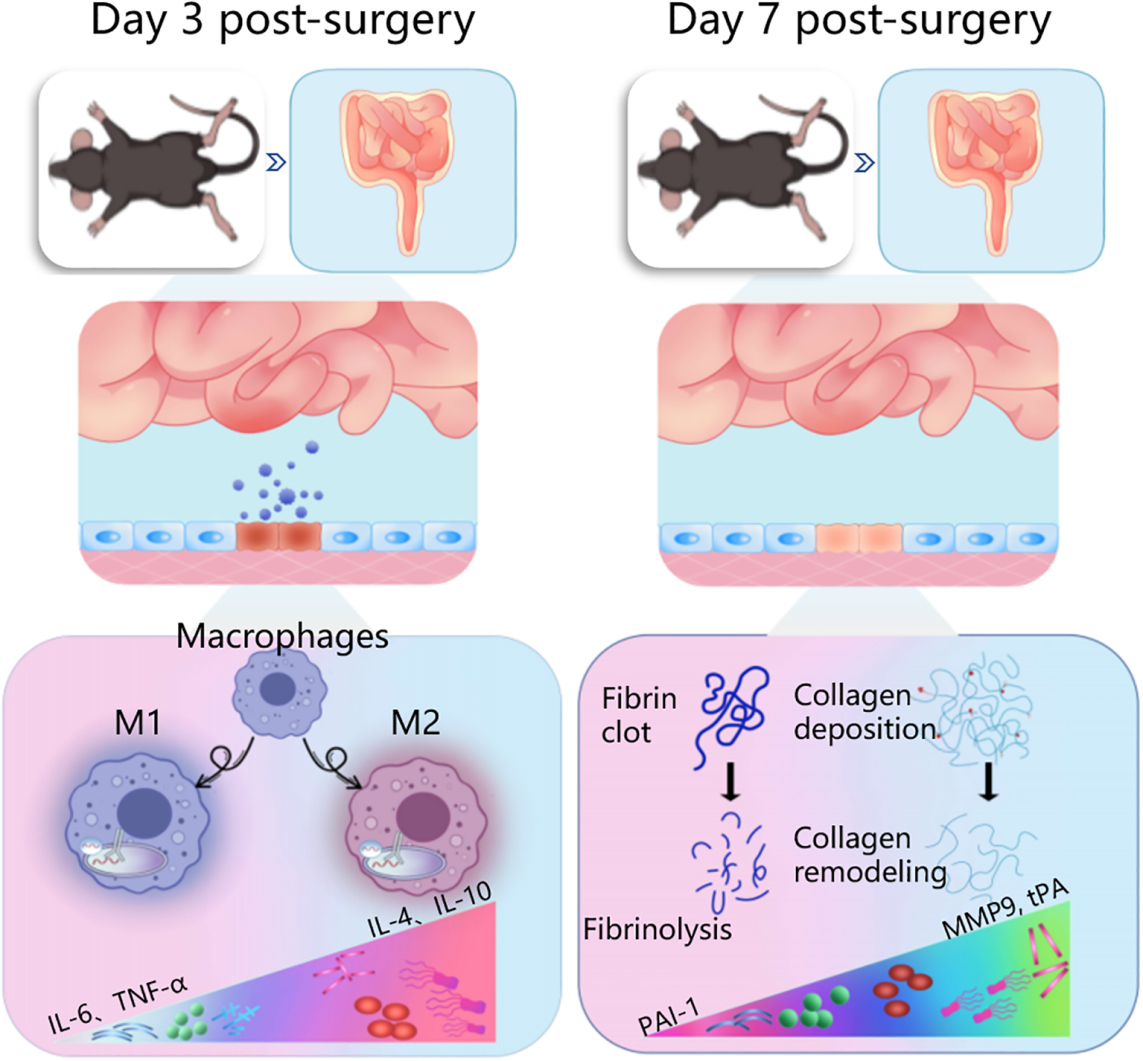
Ameliorative effects of herbal HXTF on PPA formation within a time window of either the early stage (Day 3 postsurgery) or middle stage (Day 7 postsurgery). HXTF, *HuoXueTongFu* Formula; PPA, postoperative peritoneal adhesion.

In summary, our findings demonstrated that HXTF could induce macrophage polarization-mediated anti-inflammatory reactions by increasing the phagocytic capacity of PMs and promoting the release of anti-inflammatory cytokines within 3 days after model preparation. HXTF promoted fibrinogenolysis and improved fibrinolytic activity, thereby inhibiting collagen deposition and reducing the adhesion score within 7 days after the model preparation. The cumulative results suggested that HXTF could prevent and treat PPA formation within the time window of either the early stage (Day 3 postsurgery) or middle stage (Day 7 postsurgery) (**Figure 8**). HXTF may be a promising alternative agent for the prevention and treatment of PPA.

## Data Availability

The raw data supporting the conclusions of this article will be made available by the authors, without undue reservation.
